# Dual mechanisms of peroxymonosulfate activation by a CoCuLDH/CoCuMOF composite for pollutant degradation via radical and nonradical pathways

**DOI:** 10.1038/s41598-026-64251-y

**Published:** 2026-08-08

**Authors:** Aya S. Mahmoud, Abdelrahman M. Rabie

**Affiliations:** 1 Chemistry Department, Faculty of Women, Ain Shams University, Cairo, Egypt; 2 Petrochemicals Department, Egyptian Petroleum Research Institute (EPRI), Nasr City, 11727 Cairo, Egypt

**Keywords:** CoCu-LDH@CoCu-MOF heterostructures, Advanced oxidation processes (AOPs), Peroxymonosulfate activation, Radical and non-radical degradation, Wastewater treatment, Chemistry, Environmental sciences, Materials science

## Abstract

Peroxymonosulfate (PMS)-based advanced oxidation processes have attracted considerable attention for degrading persistent organic pollutants in wastewater. However, developing efficient and stable catalysts for PMS activation remains a challenge. This study aims to design hybrid catalysts combining layered double hydroxide (LDH) and metal-organic framework (MOF) structures to enhance catalytic performance through interfacial synergy. Two bimetallic composites, CoCu-LDH@CoCu-MOF (LM) and CoCu-MOF@CoCu-LDH (ML), were synthesized and characterized using X-ray diffraction, Fourier transform infrared spectroscopy, X-ray photoelectron spectroscopy, Brunauer-Emmett-Teller analysis, scanning electron microscopy, and transmission electron microscopy. Their catalytic performance was evaluated in PMS-activated degradation of organic pollutants under optimized conditions, supported by radical quenching and reusability tests. LM showed superior catalytic activity, achieving 98.9% degradation of methylene blue (MB) within 10 min., and effectively degrading other pollutants Under the same optimized conditions, the LM/PMS system also achieved 99.6% removal of crystal violet (CV) and complete (100%) removal of malachite green (MG) within 10 min, along with 91.1% removal of p-nitrophenol (PNP) and 82.7% removal of tetracycline hydrochloride (TCH) within the same time frame, confirming the broad applicability of the system toward structurally diverse organic contaminants. Mechanistic studies indicated that singlet oxygen (^1^O_2_) was the dominant reactive species, with additional contributions from hydroxyl, sulfate, and superoxide radicals. The catalyst retained 96.9% removal efficiency after three cycles. This performance is attributed to the synergistic LDH-MOF interface, which enhances reactive species generation and provides abundant active sites for efficient PMS activation.

**Supplementary Information:**

The online version contains supplementary material available at 10.1038/s41598-026-64251-y.

## Introduction

Nowadays, inappropriately treated wastewater by the textile industry is the main cause of the global water pollution issue^1^. Even though water constitutes approximately 72% of the Earths surface, safe and dependable drinking water availability is still a major concern worldwide^2,3^. Disposing of wastewater containing dyes poses a serious risk to human health and the environment^4^. Significant amounts of highly pigmented effluent, containing a variety of persistent contaminants, are produced by textile factories^5–7^. Dyes are extensively used in massive amounts in different industrial products, including plastics, paints, leather, paper, textiles, wood, and more^8^. Regretfully, during the production and dyeing operations, significant amounts of the dyes are lost due the contamination^9^.

A heterocyclic dye with a positive charge, such as MB, finds widespread use in dyes, biostains, chemical indicators, and medicines^10^. The release of MB into water systems poses significant environmental concerns due to its persistence and toxicity, affecting both aquatic ecosystems and drinking water quality^11,12^. Various methods, such as adsorption^13^ and photodegradation^14^, chemical oxidation^15^, and biological degradation^16^, have been utilized to remove dyes from wastewater^17^. Previously, wastewater containing organic contaminants has been eliminated by conventional techniques such as flocculation, adsorption, membrane filtration, and biological treatment. However, these approaches have limitations, mostly because they are ineffective at eliminating pollutants or non-destructive, which may result in the production of secondary pollutants, such as sludge^18,19^.

Therefore, the continuous development of cost-effective and environmentally benign treatment methods is crucial for establishing efficient strategies to eliminate these persistent pollutants from dye industry effluents^9^.

Advanced oxidation processes (AOPs) are widely recognized as effective redox-based techniques capable of generating reactive species^20,21^, which play a crucial role in the efficient degradation of various pollutants in industrial wastewater systems^22,23^. AOP techniques are crucial applications for water treatment because of their efficient application, eco-friendliness, sustainability, and efficient procedures, which provide advanced ways to degrade organic pollutants^24,25^. Peroxymonosulfate (PMS)-AOP techniques generate reactive oxygen species (ROS) possessing high oxidation potentials^20,26^. Furthermore, these species are essential for degradation processes^27–29^.

In recent years, transition-metal-based heterogeneous activation has demonstrated distinct benefits compared to other approaches, offering high efficiency, straightforward operation, reduced energy demand, and convenient recovery with good reusability^30^.

Transition metals, including Co, Fe, Ni, Cu, and Mn, have attracted significant attention due to their crucial contribution to PMS activation through their redox capabilities^31,32^. Among them, Co(II) has shown outstanding catalytic performance. However, its homogeneous use often results in the leaching of metal ions into the environment, posing serious health and environmental risks. To address this issue, heterogeneous catalysts have been developed to reduce metal leaching and enhance operational stability^33^. Moreover, introducing bimetallic systems, such as Co-Cu, has proven effective in enhancing catalytic activity while minimizing ion release^34^. Copper (Cu), known for its excellent electrical conductivity, facilitates electron transfer within the catalyst and synergistically improves PMS activation^34,35^.

Metal-organic frameworks (MOFs) are crystalline porous structures formed by metal centers linked with organic ligands^36,37^. They have recently emerged as promising catalysts in sulfate radical-driven AOPs (SR-AOPs), owing to their exceptionally large surface area, abundant active sites, and remarkable catalytic efficiency^31,38^. Their well-defined structures, in which metal ions or clusters act as central nodes and organic ligands act as bridging connectors, coupled with outstanding physicochemical features including significant porosity, broad surface area, tunable metal centers and organic linkers, as well as distinctive morphology, have driven their extensive applications in areas including energy storage and conversion^38–41^. Furthermore, recent studies indicate that bimetallic MOFs exhibit superior catalytic activity compared to their monometallic counterparts, owing to the synergistic interactions between different metal centers^42^. In addition to their intrinsic catalytic potential, MOFs can serve as highly effective supports, offering an ideal platform for anchoring other functional materials such as layered double hydroxides (LDH). This promotes better dispersion and enhances the accessibility of active sites for catalytic reactions. LDHs, representative transition metal-based materials, have been widely acknowledged as highly promising catalysts. Furthermore, LDH nanoparticles have been extensively recognized as highly efficient nanostructures that integrate cost-effectiveness and environmental compatibility with excellent recyclability, superior catalytic activity, distinctive electronic configurations, and tunable structural features^43,44^. LDH is a two-dimensional material composed of positively charged cationic layers separated by interlayer anions. In recent years, LDHs have attracted significant research attention for their efficiency in dye removal from wastewater. They have been extensively applied in sulfate radical-driven AOPs due to their remarkable characteristics, including low toxicity, high structural stability, facile synthesis from low-cost raw materials, and synergistic interactions among different transition-metal ions^17,45^.

A contemporary strategy involves the integration of LDHs with MOFs to construct hybrid catalytic systems that synergistically combine the intrinsic advantages of both materials. This coupling enhances PMS activation efficiency, thereby promoting the effective degradation of organic pollutants in aqueous media. In particular, the CoCu-LDH@CoCu-MOF system provides a promising platform to exploit the complementary catalytic properties of both components, resulting in improved oxidative performance and enhanced removal of dye contaminants from wastewater^46^.

Despite the individual advantages of MOFs and LDHs, both materials are still constrained by inherent limitations, including suboptimal electron transfer dynamics and restricted accessibility of active sites. To address these challenges, the construction of LDH-MOF heterostructures has recently emerged as an effective strategy to promote interfacial charge transfer, increase active site exposure, and enhance overall catalytic performance^46,47^. Nevertheless, the underlying mechanistic aspects of PMS activation in such hybrid systems, particularly the interplay between radical and non-radical pathways, remain insufficiently understood.

In this work, two hybrid composites, CoCu-LDH@CoCu-MOF and CoCu-MOF@CoCu-LDH, were synthesized and evaluated for the catalytic degradation of MB in the presence of PMS. The effects of key operational parameters, including catalyst dosage, PMS concentration, initial dye concentration, and solution pH, were systematically investigated. In addition, the catalytic mechanism was elucidated through identification of ROS, highlighting the concurrent contributions of radical and non-radical pathways.

Overall, this study not only demonstrates high catalytic efficiency of the synthesized heterostructures but also provides deeper insight into PMS activation mechanisms in LDH-MOF hybrid systems. These findings contribute to the rational design of advanced catalysts for sustainable and efficient wastewater treatment applications.

## Materials and methods

### Materials

Cobalt nitrate hexahydrate (Co(NO_3_)_2_•6H_2_O, ≥ 99.0%), Cupric nitrate trihydrate (Cu(NO_3_)_2•_3H_2_O, ≥ 99.0%), N, N-dimethylformamide (DMF, ≥ 99.5%), terephthalic acid (H_2_BDC, ≥ 99.0%), pure ethanol (EtOH), methanol (MeOH), isopropyl alcohol (IPA) C_3_H_8_O were provided from Sigma Aldrich, *p*-benzoquinone (PBQ, ≥ 99.0%), L-histidine (LH, ≥ 98.0%), sodium hydroxide (NaOH), hydrochloric acid (HCl), sodium carbonate (Na_2_CO_3_), Sodium sulfate (Na_2_SO_4_.7H_2_O), sodium nitrate (NaNO_3_), sodium chloride (NaCl), Methylene blue (MB), Crystal violet (CV), Malachite green (MG), *P*-nitrophenol (PNP), and tetracycline hydrochloride (TCH) were supplied by Loba Chemi Company. Every chemical and reagent utilized throughout this study was of analytical quality and didn’t require further purification. This study was conducted using deionized water (DI). For simplicity, the prepared samples were denoted as follows: CoCu-LDH (L), CoCu-MOF (M), CoCu-MOF@CoCu-LDH (ML), and CoCu-LDH@CoCu-MOF (LM).

### Preparation of CoCu-LDH (L)

For the synthesis of CoCu-LDH, Co (NO_3_)_2_.6H_2_O (6.88 mmol, 2.0 g) and Cu(NO_3_)_2_.3H_2_O (2.13 mmol, 0.5 g) were dissolved in a solvent mixture of 15 mL EtOH and 35 mL deionized water under continuous stirring. Subsequently, urea (24.97 mmol, 1.5 g) was added, and the solution was continuously stirred for 30 min to obtain a homogeneous mixture. The precursor solution was then transferred into a Teflon-lined stainless-steel autoclave and heated for 12 h at 120 °C. After natural cooling to room temperature, the obtained precipitate was collected by centrifugation, thoroughly washed with EtOH and DI water, then dried for 12 h at 60 °C^48,49^, and then kept for the next study.

### Preparation of CoCu-MOF (M)

For the synthesis of CoCu-MOF, (4.22 mmol, 1.23 g) of Co(NO_3_)_2_·6H_2_O, (4.22 mmol, 1.02 g) of Cu(NO_3_)_2_·3H_2_O, and (8.45 mmol, 1.40 g) of H_2_BDC were dissolved in a mixed solvent containing 28 mL DI and 40 mL DMF. The solution was stirred for 30 min to achieve complete homogenization. Subsequently, the obtained solution was transferred into a Teflon-lined stainless-steel autoclave and maintained for 8 h at 120 °C. After cooling to ambient temperature, the suspension was centrifuged and repeatedly washed with DMF and DI water to remove residual ions and unreacted ligands. The final product was dried for 7 h at 70 °C and preserved for subsequent use.

### Preparation of CoCu-MOF@ CoCu-LDH (ML)

For preparation of ML, 0.3 g of prepared L was dispersed into a mixed solution containing (1.4 mmol, 0.41 g) of Co(NO_3_)_2_.6H_2_O, (1.4 mmol, 0.338 g) of Cu(NO_3_)_2_.3H_2_O, and (2.8 mmol, 0.47 g) of H_2_BDC, which were dissolved in 28 mL of deionized water (DI) and 40 mL of DMF Then the obtained suspension was subjected to the same synthesis procedure as that used for M preparation.

### Preparation of CoCu-LDH@CoCu-MOF (LM)

**Fig. 1:**
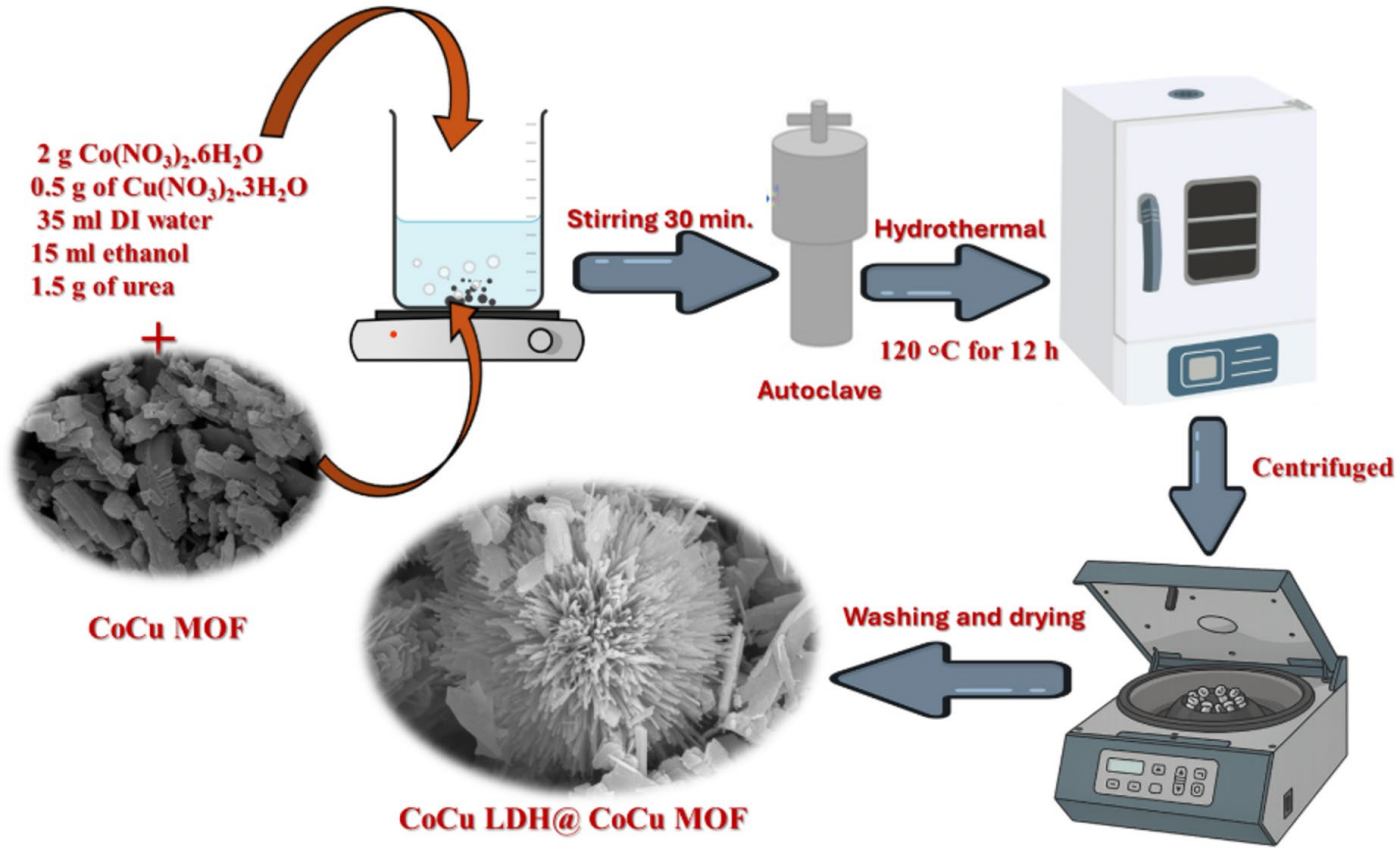
Scheme of preparation of CoCu-LDH @ CoCu-MOF (LM).

The LM composite was synthesized by adding 0.3 g of the prepared M into a mixed solution containing (0.57 mmol, 0.166 g) of Co(NO_3_)_2_.6H_2_O, (2.80 mmol, 0.677 g) of Cu(NO_3_)_2_.3H_2_O and (8.33 mmol, 0.50 g) of urea (CH_4_N_2_O), which were subsequently dispersed in a mixed solvent of 35 mL deionized water and 15 mL EtOH, Afterward, the same technique for L preparation was carried out as illustrated in Fig. 1. Details of characterization techniques and instrument specifications are provided in the supplementary file.

### The catalytic performance

Initially, the catalytic performance of L, M, ML, and LM was assessed for MB degradation in the presence of PMS to determine the most efficient catalyst under identical conditions. To initiate the catalytic degradation experiment, 2.5 mg of catalyst was introduced into 50 mL of MB solution (20 mg L^− 1^) and magnetically stirred for 30 min to establish adsorption–desorption equilibrium. The reaction was initiated by introducing 10 mg of PMS, followed by continuous stirring at 25 °C for 60 min. At predetermined time intervals, 2 mL aliquots were withdrawn from the reaction mixture and immediately quenched with 0.1 mL of 0.1 M sodium thiosulfate (Na_2_S_2_O_3_) solution to quench residual PMS and stop further oxidation reactions. The samples were then filtered (0.24 μm) and analyzed by UV-Vis spectrophotometry at 664 nm. Moreover, the effects of catalyst dose, PMS dose, initial pH, initial dye concentration, and coexisting ions were studied.

For the coexisting-ion experiments, each inorganic anion (Cl^−^, NO_3_^−^, SO_4_^2−^, and CO_3_^2−^) was introduced individually as the corresponding sodium salt at a concentration of 20 mM into the standard reaction system (2.5 mg of LM catalyst, 10 mg PMS, 50 mL of 20 mg L^− 1^ MB, pH 7). For the radical scavenging experiments, (MeOH, 1 M) and (IPA, 1 M) were used as scavengers for ^•^OH/SO_4_^•-^ and ^•^OH, respectively, while p-benzoquinone (PBQ, 2 mM) and (LH, 10 mM) served as O_2_^•-^ and ^1^O_2_ scavengers, respectively. All scavengers were added to the reaction mixture 5 min prior to PMS introduction.

ROS were identified through quenching experiments using MeOH, IPA, PBQ, and LH to trap ^•^OH, SO_4_^•-^, O_2_^•-^, and ^1^O_2_, respectively. All experiments were conducted in triplicate, and the average values along with corresponding error bars were presented. Furthermore, the catalytic performance was also examined for the degradation of other toxic and carcinogenic pollutants such as MG, CV, PNP, and TCH. For the reusability assessment, the catalyst was recovered from the reaction mixture, carefully rinsed with EtOH and distilled water, then dried at 60 °C before reuse in consecutive degradation cycles.

## Results and discussion

### Characterization

**Fig. 2:**
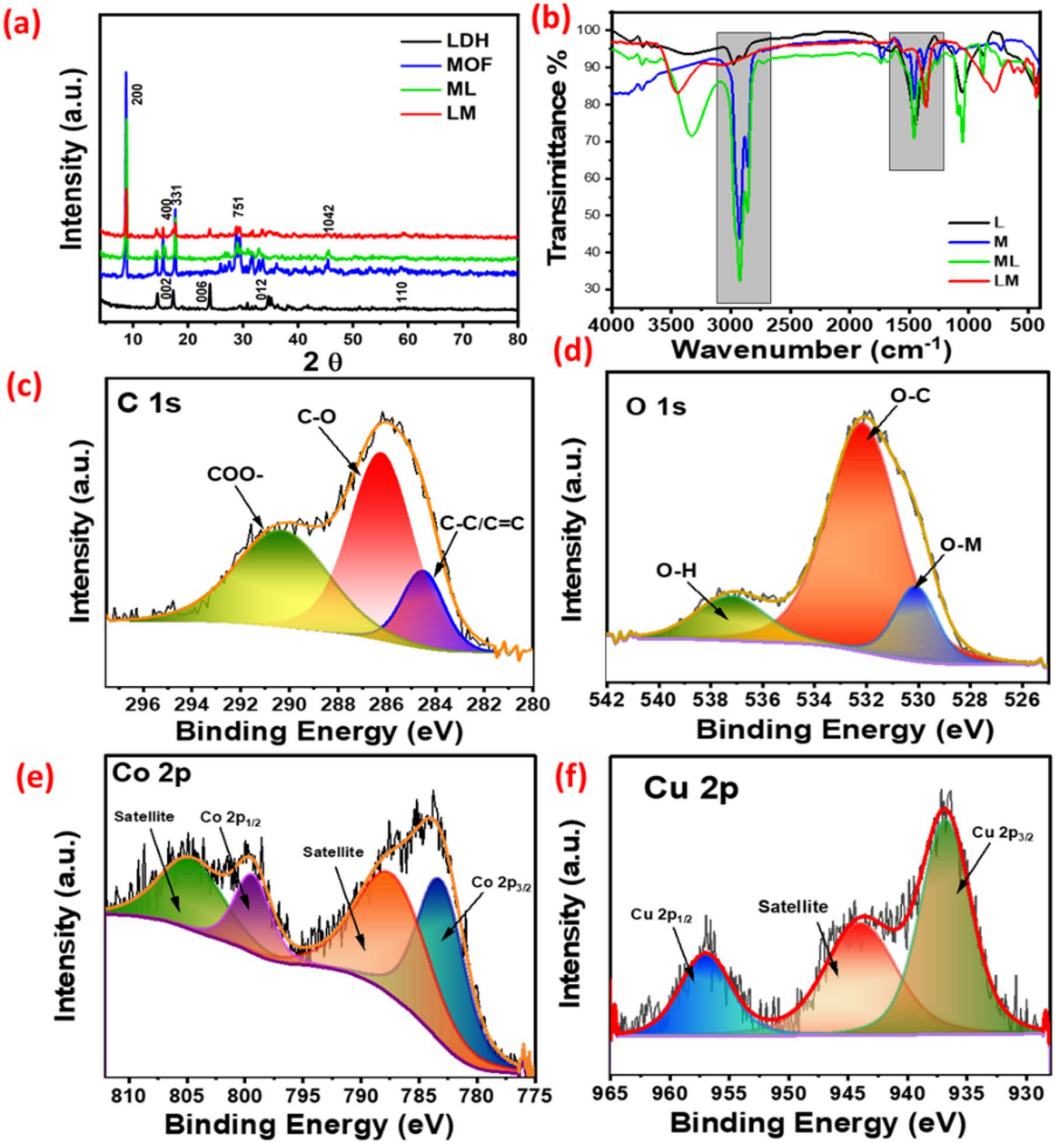
(**a**) XRD patterns, (**b**) FTIR spectra, and XPS spectra of LM: (**c**) C1 s, (**d**)  O1s, (e) Co 2p, and (**f**) Cu 2p regions.

The XRD patterns of the pristine L, M, and their binary composites ML and LM are shown in Fig. 2a. The more discernible LDH-related reflections observed at higher angles, particularly around 2θ ≈ 22–23°, 34–35°, and near 60°, can be attributed to the higher-order basal/non-basal planes of the LDH phase, supporting the formation of a brucite-like layered hydroxide structure in the prepared CoCu-LDH sample, in agreement with the adopted LDH reference pattern, JCPDS No. 51-0045^50^. On the other hand, the CoCu-MOF sample displays a set of sharp and intense diffraction peaks, confirming its high crystallinity. The strong reflection at approximately 2θ ≈ 8.7° is assigned to the (200) plane of the MOF phase, while the peaks observed around 15° and 17–18° are assigned to the (400) and (331) reflections, respectively. Additional MOF-related peaks appearing at approximately 29–30° and 45–46° correspond to higher-index planes, such as (751) and (1042), according to the MOF reference pattern, JCPDS No. 50–2286^51^. These well-defined peaks confirm the successful formation of the CoCu-MOF crystalline framework. For the ML composite, both MOF and LDH related diffraction features are observed, indicating the coexistence of the two phases without complete structural collapse during the second hydrothermal growth step. The MOF peaks, especially those located at approximately 8.7°, 15°, 17–18°, 29–30°, and 45–46°, remain dominant in ML, suggesting that the MOF phase contributes significantly to the diffracting volume and is effectively grown on the pre-formed LDH surface. Meanwhile, the LDH-related reflections become less pronounced, which may be due to partial coverage of the LDH domains by MOF crystallites and/or overlap with the stronger MOF diffraction peaks. In contrast, the LM composite shows relatively more evident LDH-related contributions together with retained MOF reflections, indicating that LDH was successfully deposited onto the pre-formed MOF structure. The reduction in intensity and broadening of some MOF reflections in LM may be attributed to partial surface restructuring of the MOF under the alkaline LDH growth conditions, while the persistence of the main MOF peaks confirms that the framework was not completely destroyed. Overall, the XRD results support the successful preparation of the two hybrid MOF/LDH heterostructures through sequential growth. The simultaneous presence of characteristic reflections from both components, together with changes in peak intensity and broadening compared with the pristine phases, suggests interfacial integration between the LDH and MOF domains.

The FTIR spectra of L, M, ML, and LM samples exhibit distinctive absorption bands corresponding to the characteristic functional groups of LDH and MOF structures, as shown in Fig. 2b. A wide absorption band observed within 3400–3450 cm^− 1^ is characteristic of O-H stretching vibrations, indicating the presence of hydroxyl groups and interlayer water molecules, confirming the hydrophilic nature of the LDH framework^52^. In addition, the peak observed around 1630 cm^− 1^ is associated with the bending vibration of adsorbed water molecules, further supporting the presence of interlayer water within the structure^53^. The absorption bands located in the region of 2850–2950 cm^− 1^, which are more pronounced in M, ML, and LM samples, are assigned to C-H stretching vibrations of the organic linkers, indicating the successful incorporation of the MOF component^54^. In the low-frequency region (500–800 cm^− 1^), the absorption bands are typically associated with metal-oxygen (M-O) stretching vibrations, corresponding to the Co-O and Cu-O bonds^55,56^. Additionally, the MOF-containing samples display minor features within 900–1200 cm^− 1^ attributed to C-O stretching and M-O-C linkage vibrations within the organic-inorganic coordination network^57^. The hybrid composites (ML and LM) clearly combine the spectral features of both parent materials. Notably, the enhanced intensity and slight shifts of the O-H and M-O bands indicate strong interfacial interactions between the LDH and MOF phases, confirming successful hybridization. Such structural integration is expected to increase active site density and improve catalyst stability, which directly correlates with the enhanced catalytic activity observed in the degradation studies.

XPS analysis of ML composite confirms the successful coordination of bimetallic species with organic ligands, revealing distinct chemical states and synergistic interactions. The XPS spectral survey in supplementary materials (Fig. S1) evidences the coexistence of Cu, Co, O, and C elements, verifying the expected elemental composition of the hybrid catalyst. The C 1 s spectrum in Fig. 2c shows several distinct contributions. The dominant peak at approximately at ~ 284.8 eV is attributed to C-C/C = C bonds from the organic framework^17,58^. The component at ~ 286.2 eV is assigned to C-O groups, indicating aromatic carbon structures and oxygen-functionalized ligands. In comparison, the peak near 288.8 eV represents carboxylate COO^−^ groups, which are typical in MOF linkers^17,59^. These carbon functionalities confirm the incorporation of the organic framework and its interaction with the LDH phase. The O 1 s spectrum in Fig. 2d deconvolutes into O-M (M = Co/Cu) at 530 eV, confirming the presence of Co-O and Cu-O coordination within the hybrid structure, and O-C at 532 eV, confirming metal-oxygen coordination and organic-inorganic hybridization^17,58^. The third absorption peak is indicative of surface-adsorbed water molecules on the material^27^. Cu 2p spectrum in Fig. 2f exhibits a main Cu 2p₃_/_₂ peak at ~ 937 eV and a Cu 2p_1/2_ peak at ~ 957 eV, characteristic of Cu(II) species^58^. The intense shake-up satellites observed at ~ 944 eV confirm the dominance of Cu^2+^, as such features are absent in Cu^+^ or Cu⁰ states. This indicates that copper mainly exists in a divalent oxidation state in the composite^59^. While the Co 2p spectrum in Fig. 2e shows Co^2+^ signatures at 783 eV (2p_3/2_) and 799 eV (2p_1/2_), validating stable cobalt coordination with satellites near ~ 787 eV and ~ 804 eV. These findings demonstrate the integration of mixed-valence metals and organic frameworks, enhancing electrocatalytic properties for sensing applications^58,59^. The coexistence of Co^2+^ and Co^3+^ suggests a mixed-valence state, which enhances redox cycling and catalytic activity in PMS activation.

**Fig. 3:**
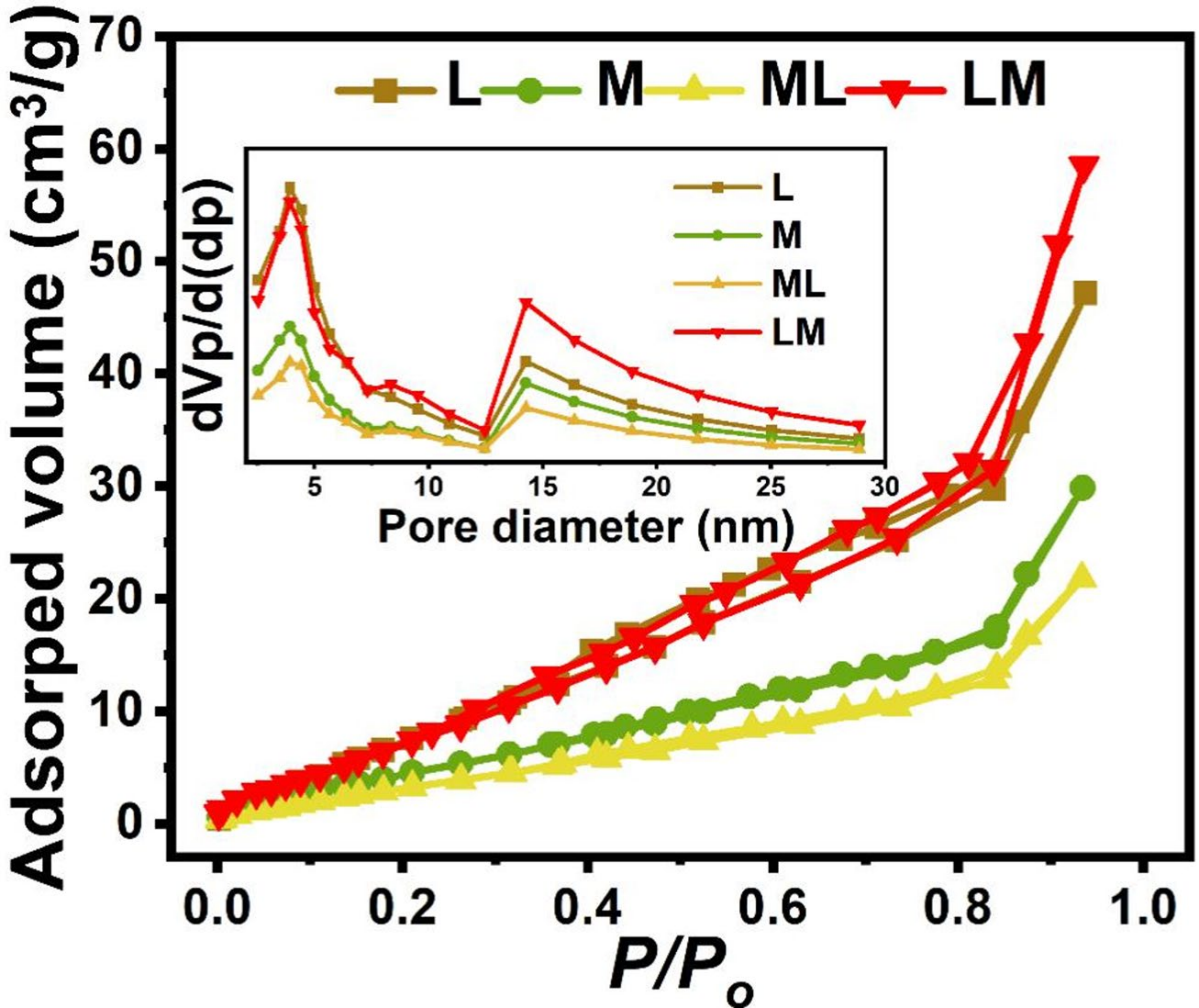
BET, pore volume, and pore size distribution of the prepared materials.

The nitrogen adsorption–desorption isotherms and BET analyses presented in Fig. 3 provide comprehensive insights into the surface architecture and porosity evolution of the synthesized CoCu-based materials (L, M, ML, and LM). All samples exhibit a typical type IV adsorption isotherm accompanied by a pronounced H3 hysteresis loop within the relative pressure (*P/P₀*) interval of 0.4–0.9, confirming the presence of mesoporous structures with slit-like pores typical of layered composites as defined by IUPAC. The adsorption branch corresponds to the progressive filling of mesopores through capillary condensation, whereas the desorption branch signifies the release of physisorbed nitrogen molecules, revealing the degree of pore connectivity and the structural stability of the frameworks. Quantitatively, the specific surface area (S_BET_) values follow the order L (50.61 m^2^ g^− 1^) > LM (46.26 m^2^ g^− 1^) > M (24.69 m^2^ g^− 1^) > ML (18.59 m^2^ g^− 1^), as shown in supplementary materials Table S1.

This decreasing trend indicates that integrating the MOF phase into the LDH matrix LDH@MOF (LM) effectively preserves the lamellar configuration and prevents layer collapse, whereas the reverse configuration, MOF@LDH (ML), partially blocks internal pores, thereby reducing accessible surface area. A similar pattern is observed in the total pore volume (LM: 0.00985 > L: 0.008 > M: 0.00496 > ML: 0.00367 cm^3^ g^− 1^), highlighting that the LM heterostructure provides a more open and accessible porous network favorable for mass transport and enhanced PMS activation kinetics. The average pore diameters, ranging between 3.85 and 3.94 nm, confirm the dominance of mesoporous channels that facilitate efficient diffusion and utilization of active sites. The BJH pore size distribution curves presented in the inset of Fig. 3 reveal a bimodal pore size distribution for all samples, with two distinct pore populations. The primary distribution is centered at ~ 4–5 nm and can be attributed to the intrinsic mesoporosity associated with the CoCu-LDH layers and the porous CoCu-MOF framework. A secondary pore population centered at ~ 15 nm is particularly evident for the LM composite and is attributed to textural mesoporosity arising from interparticle voids generated during the hierarchical assembly of LDH nanosheets and MOF crystals. These larger mesopores are expected to facilitate the diffusion and mass transfer of reactants and oxidants to the active sites, while the smaller mesopores provide a high surface area for adsorption and catalytic reactions. Therefore, the hierarchical bimodal pore structure of LM likely contributes to its enhanced catalytic performance compared with the individual L and M materials. Collectively, the data in Fig. 3 and Table S1 validate the successful formation of a structurally synergistic LM composite in which the two-dimensional LDH layers contribute hierarchical accessibility. At the same time, the three-dimensional MOF framework provides additional porosity, thereby generating an optimized surface environment for improved electron transfer, adsorptive interactions, and catalytic PMS activation efficiency in AOPs.

**Fig. 4:**
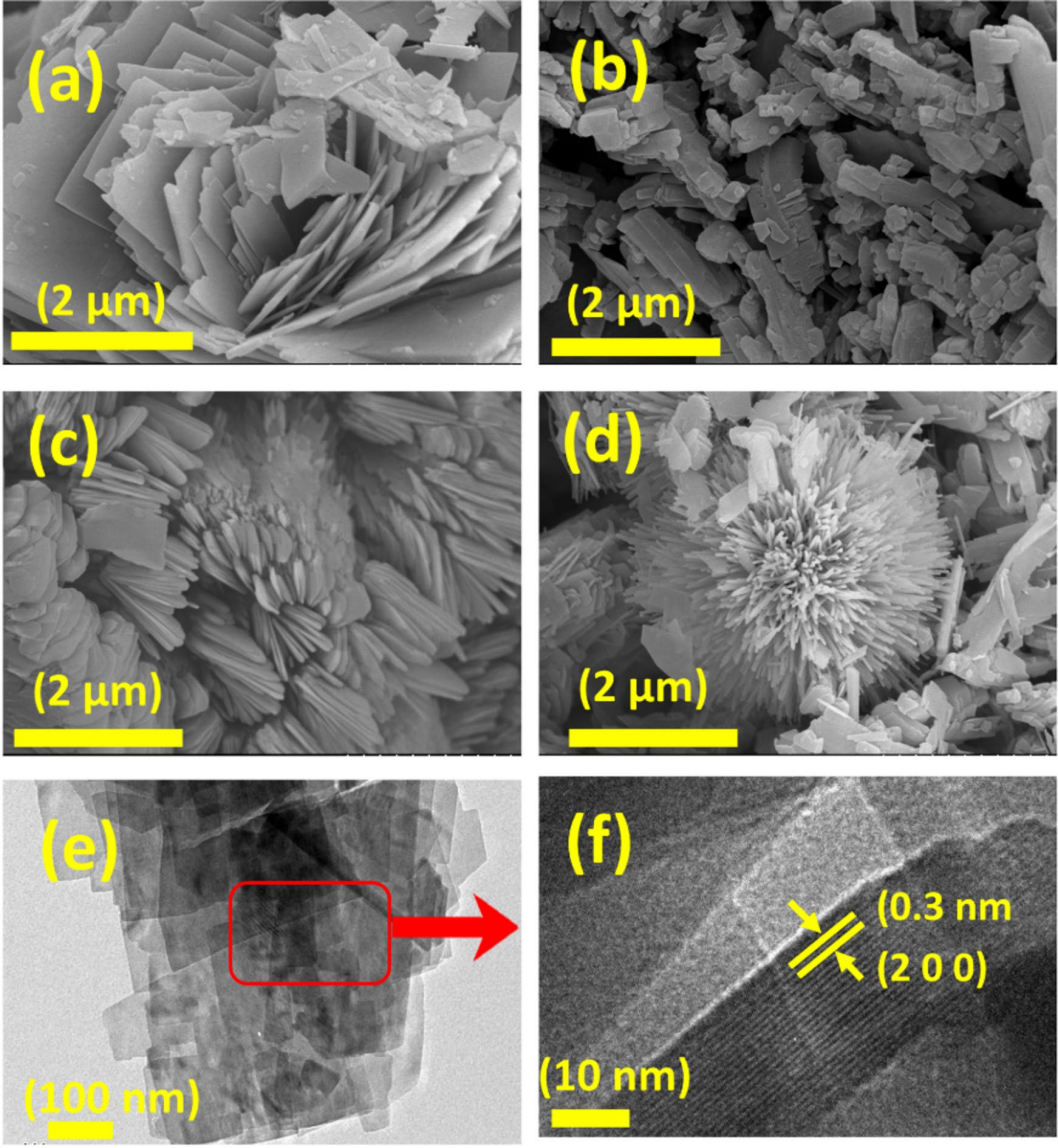
SEM images of (**a**) L, (**b**) M, (**c**) ML, and (**d**) LM, and TEM images (**e**, **f**) of LM.

The SEM images clearly demonstrate the distinct morphologies of the synthesized samples. Pure L in Fig. 4a exhibits stacked platelet-like nanosheets, a typical feature of layered double hydroxides. In contrast, the M in Fig. 4b displays irregular polyhedral particles, consistent with the crystalline structures of Cu-based metal–organic frameworks. For the ML composite in Fig. 4c, MOF particles are deposited on the LDH surface, leading to partial coverage that may limit the full accessibility of LDH active sites. On the other hand, the LM composite in Fig. 4d forms a flower-like hierarchical structure, in which LDH nanosheets grow uniformly on the MOF particles, providing a rougher texture, larger surface area, and better dispersion of catalytic sites. EDX elemental mapping confirming the uniform distribution of Co, Cu, O, and C throughout the LM composite is provided in the supplementary material Fig. S2. These observations are further confirmed by TEM analysis in Fig. 4e and f, where thin LDH nanosheets are anchored onto the MOF framework, forming abundant interfaces. The HRTEM micrograph displays distinct lattice fringes with an interplanar distance of about 0.30 nm, assignable to the (200) crystal plane of the LDH structure, which confirms the high crystallinity and intimate contact between LDH and MOF. Such hierarchical integration is expected to facilitate charge transfer and enhance pollutant adsorption, ultimately boosting catalytic efficiency in PMS activation.

### Catalytic activity

**Fig. 5:**
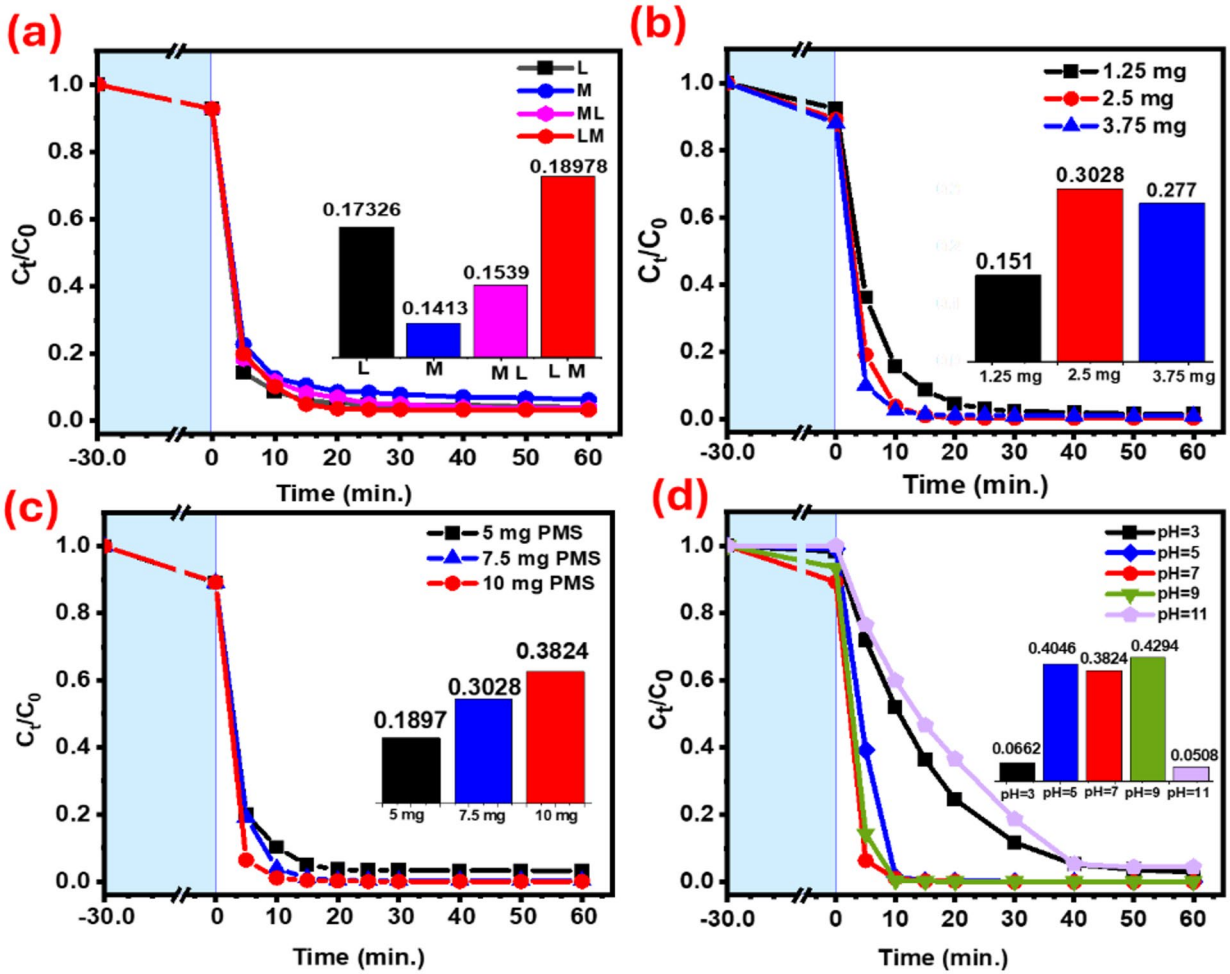
(**a**) Comparison of the catalytic performances of L, M, ML, and LM for MB degradation, (**b**): The effect of different catalyst doses, (**c**): The effect of PMS dose, and (**d**): The effect of pH for MB degradation.

To establish adsorption-desorption equilibrium, all reaction systems, the synthesized catalyst, and the MB dye were mixed and stirred for 30 min before adding PMS. As shown in Fig. 5a, the catalytic performances of L, M, ML, and LM were compared for MB degradation under identical conditions (2.5 mg catalyst, 5 mg PMS, 20 mg L^− 1^ dye, 50 mL). All catalysts exhibited rapid dye degradation within the initial minutes of reaction; however, the LM composite demonstrated the highest activity, achieving nearly complete MB removal in the shortest time, followed by L, ML, and M. Pseudo-first-order kinetic modelling confirmed this results, providing the calculated rate constant values 0.18978, 0.17326, 0.1539, and 0.14135 min^− 1^ for LM, L, ML, and M, respectively. Additional pseudo-first-order kinetic fitting plots for all investigated experimental parameters are provided in Fig. S4 of the supplementary material. The superior performance of LM arises from the synergistic interaction between the LDH and MOF phases, which enhances active site accessibility, facilitates electron transfer, and promotes PMS activation, thereby generating ROS (^**•**^OH, ^1^O_2_, SO_4_^•-^, and O_2_^•-^). Moreover, these observations align with previous reports on bimetallic hybrid catalysts exhibiting enhanced efficiency in AOPs^60–62^, confirming LM as the most effective system for dye degradation under the tested conditions.

To delineate the individual contributions of adsorption, direct PMS oxidation, photolysis, and catalytic PMS activation to the overall MB removal, five control experiments were conducted under identical conditions (2.5 mg catalyst, 10 mg PMS, 50 mL of 20 mg L^− 1^ MB, pH 7). The corresponding UV–Vis spectra together with the removal efficiency inset, are presented in the supplementary materials Fig. S3. MB alone showed negligible absorbance change at 664 nm, confirming its stability under ambient laboratory conditions. The MB + PMS system achieved only 10.8% removal, indicating the limited oxidative ability of PMS in the absence of catalytic activation. The MB + LM system exhibited 15.1% removal, which was attributed to physical adsorption of MB onto the catalyst surface. In contrast, the LM/PMS system achieved 98.9% MB removal, demonstrating the excellent catalytic performance of the catalyst toward PMS activation. Notably, the LM/PMS system under dark conditions achieved 98.7% MB removal, which is nearly identical to the value obtained under ambient laboratory light. This result indicates that photocatalytic effects do not contribute significantly to the degradation process and confirms that the observed catalytic activity originates primarily from heterogeneous PMS activation on the LM catalyst surface.

The effect of catalyst dosage was examined by varying the catalyst weight from 1.25 to 3.75 mg while maintaining 7.5 mg PMS and 20 mgL^− 1^ MB at neutral pH, as investigated in Fig. 5b. Increasing the catalyst dosage from 1.25 to 2.5 mg significantly accelerated MB degradation, achieving nearly complete removal with 98.9% within 10 min only at 2.5 mg. This improvement results from a greater number of available active sites, which enhances PMS decomposition and radical generation. However, further increasing the catalyst mass to 3.75 mg reduced degradation efficiency due to particle aggregation, which limits surface area and hinders contact between reactive radicals and dye molecules^61,62^. Kinetic analysis supported these findings, with the rate constant reaching 0.3028 min^− 1^ at 2.5 mg, decreasing slightly to 0.277 min^− 1^ at 3.75 mg, and dropping to 0.151 min^− 1^ at 1.25 mg. The decline at low dosage results from insufficient active sites. In comparison, the decline at 3.75 mg arises from agglomeration-induced limitations in mass transfer and electron mobility. These results indicated that in AOPs, a catalyst weight of 2.5 mg is optimal for enhancing the rate of MB degradation by balancing active site availability and particle dispersion, in addition to improving degradation kinetics.

The influence of PMS concentration (5–10 mg) on MB degradation was also investigated using 2.5 mg of catalyst dispersed in 50 mL of 20 mgL^− 1^ of MB solution at pH 7, as illustrated in Fig. 5c. Increasing the PMS dosage from 5 to 10 mg markedly enhanced the degradation rate, achieving nearly complete decolorization within 10 min at 10 mg PMS. Pseudo-first-order kinetics confirmed this enhancement, as the rate constant exhibited a noticeable rise from 0.1897 to 0.3824 min^− 1^. The higher PMS concentration promotes the formation of additional ROS (^1^O_2_, ^**•**^OH, SO_4_^•–^, and O_2_^•–^), which accelerate oxidative cleavage of MB molecules^61,62^. Therefore, 10 mg PMS is identified as the optimal oxidant dosage for efficient dye degradation under the studied conditions.

The influence of pH on MB degradation using the LM catalyst and PMS was evaluated at different values of pH (3, 5, 7, 9, and 11). As shown in Fig. 5d, the degradation efficiency was significantly enhanced under neutral to slightly acidic or basic conditions, with the highest activity observed at pH 5–9. This suggests that the system performs optimally near neutral pH (pH 7) without the need for additional pH adjustment using reagents such as NaOH or HCl. Under these conditions, the effective generation of ROS, including ^1^O_2_, ^**•**^OH, SO_4_^•–^, and O_2_^•–^ promotes efficient degradation of organic pollutants^63^. At strongly acidic pH (pH = 3), the catalytic efficiency decreased markedly as a result of sulfate radical (SO_4_^•–^) scavenging by excess H^+^ ions and the instability of PMS under acidic conditions^64^. Similarly, at highly alkaline pH (pH = 11), a decline in degradation performance was observed, likely caused by excessive PMS decomposition and the formation of non-reactive species such as SO_5_^−2^, as well as possible catalyst deactivation^65,66^. Overall, these results confirm that neutral conditions (pH ≈ 7) provide an optimal environment for PMS activation and efficient MB degradation in the LM/PMS system.

**Fig. 6:**
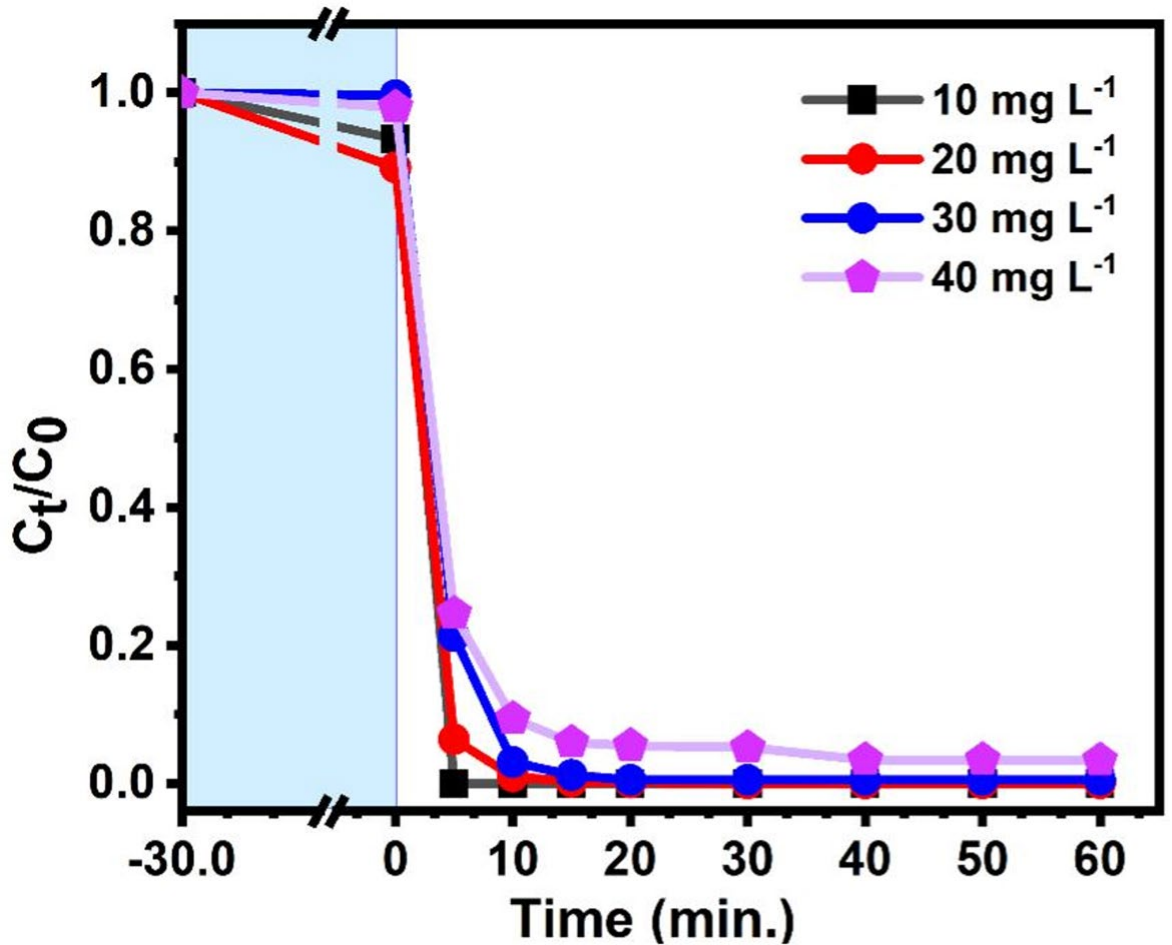
The effect of initial dye concentration at optimum conditions.

The impact of initial MB dye concentrations (10, 20, 30, and 40 mgL^− 1^) on the degradation performance of the LM catalyst in the presence of 10 mg PMS and 2.5 mg catalyst was examined. As depicted in the C_t_/C_₀_ versus time plot in Fig. 6, an increase in the dye concentration resulted in a gradual decline in degradation efficiency. At 10 and 20 mgL^− 1^, the complete degradation occurred within minutes, while at 40 mgL^− 1^, the process was significantly slower, and residual dye remained after 60 min. This inverse correlation can be attributed to the fixed number of reactive radicals generated from PMS activation. At lower dye concentrations, the number of radicals is sufficient to rapidly oxidize all dye molecules. However, at higher concentrations, the radicals become relatively insufficient compared to the number of dye molecules, resulting in incomplete degradation and slower kinetics^67,68^.

### The effect of co-existing ions on MB degradation

**Fig. 7:**
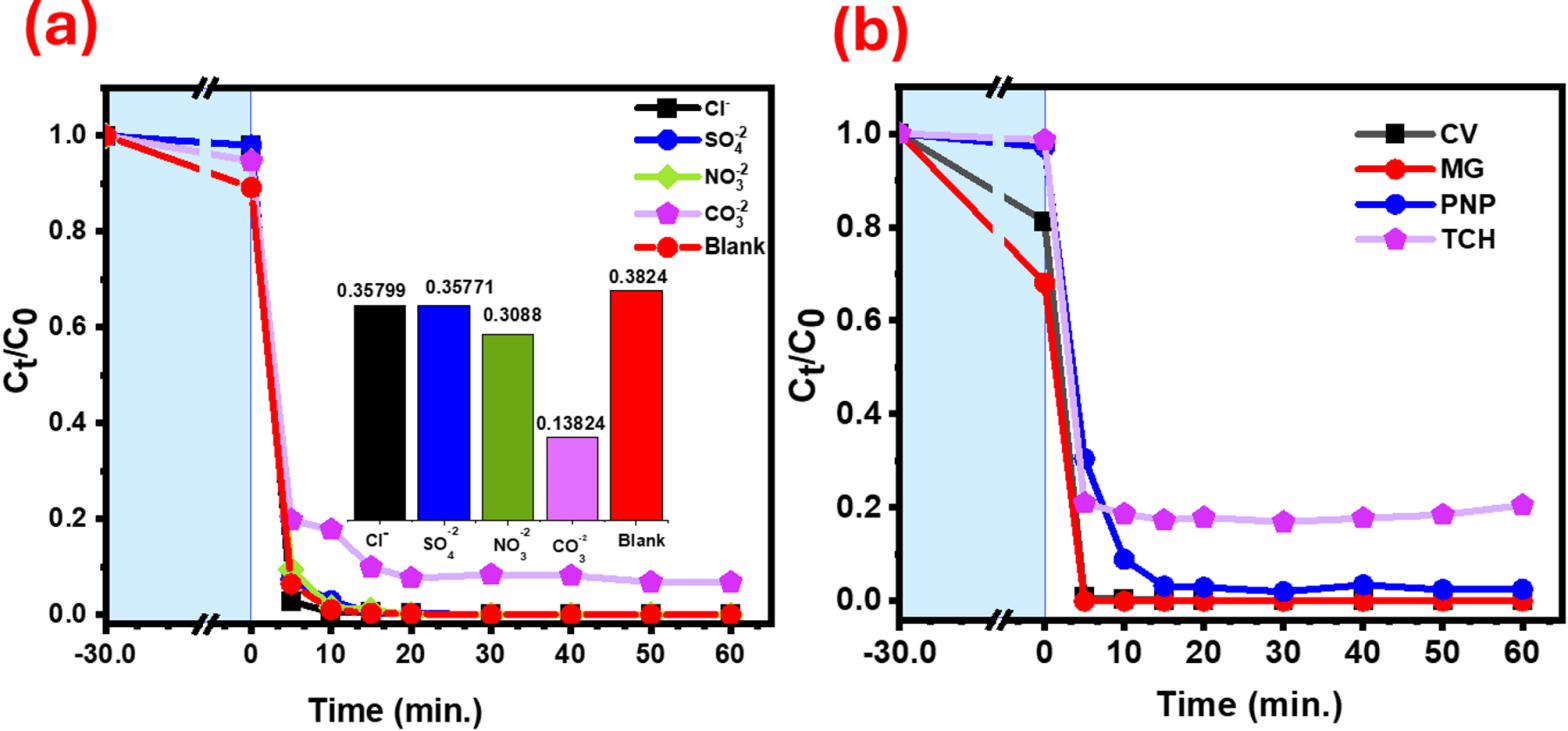
(**a**): The effect of Co-existing Ions on MB degradation at catalyst weight = 2.5 mg, MB = 20 mgL^− 1^, pH = 7, and PMS = 10 mg, (**b**): Degradation of different organic pollutants.

Under optimized conditions (2.5 mg catalyst, 10 mg PMS, 50 mL of 20 mgL^− 1^ MB, pH 7), the influence of typical inorganic anions (Cl^−^, NO_3_^−^, SO_4_^2−^, and CO_3_²^−^) on the degradation of MB dye was investigated to simulate real water matrices. The results in Fig. 7a showed minor decreases in degradation rates with Cl^−^, SO_4_²^−^, and NO_3_^−^ (k = 0.3579, 0.3577, and 0.3088 min^− 1^), respectively, while a significant decline was observed with CO_₃_²^−^ (k = 0.1375 min^− 1^), compared to the blank (k = 0.3824 min^− 1^). These variations are attributed to the ions’ interactions with reactive radicals. Cl^−^ and NO_3_^−^ weakly scavenge radicals or form secondary oxidants with lower activity, while SO_4_^2−^ may even regenerate SO_4_^2−^ radicals, partially compensating for radical loss^69,70^. In contrast, CO_3_^2−^ strongly scavenges both SO_4_^•−^ and ^•^OH, forming less reactive CO_3_^•-^, and increases ionic strength, which may hinder PMS-catalyst interactions, explaining the pronounced inhibition^70^. The competitive adsorption between the introduced inorganic anions and MB at the LDH component of the catalyst represents a complementary inhibition mechanism. The hydroxide layers of the CoCu-LDH component can interact with inorganic anions through surface complexation and anion exchange, thereby altering the adsorption environment and reducing the accessibility of MB molecules to the catalytically active Co and Cu centers. This effect is supported by the pre-reaction equilibration data shown in Fig. 7a, where the presence of CO_3_^2−^ produces the most pronounced influence on MB adsorption behavior, consistent with the strong affinity of carbonate ions toward LDH structures and their generally reported position in the anion exchange selectivity series (CO_3_^2−^ > SO_4_^2−^ > Cl^−^ ≈ NO_3_^−^)^71^. In addition, CO_3_^2−^ can effectively scavenge both SO_4_^•−^ and ^•^OH radicals, leading to the formation of the less reactive CO_3_^•−^ species^22^. Therefore, the combined effects of competitive adsorption and radical scavenging account for the significantly greater inhibition observed in the presence of carbonate ions compared with the other investigated anions.

### Degradation of different organic pollutants

Under optimized conditions (2.5 mg catalyst, 10 mg PMS), the LM/PMS system was evaluated for degrading four common organic pollutants: crystal violet CV, malachite green (MG), p-nitrophenol (PNP), and tetracycline hydrochloride (TCH), representing dyes, phenols, and antibiotics. As shown in Fig. 7b, CV, MG, and PNP were nearly fully degraded within 10 min, demonstrating strong interaction with generated radicals. Among the investigated pollutants, MG exhibited the highest adsorption capacity toward the LM catalyst prior to PMS addition. This behavior can be attributed to its highly conjugated triphenylmethane structure, which promotes strong π-π interactions with the aromatic terephthalate (BDC) linkers of the CoCu-MOF component^72^. In addition, the cationic nature of MG may contribute to favorable electrostatic interactions with the catalyst surface under the experimental conditions. The combined effect of these interactions likely enhances the affinity of MG toward the LM catalyst, resulting in higher adsorption compared with the other investigated pollutants.TCH also degraded, but at a slower rate. Despite this, the system showed reasonable efficiency in removing TCH and still reflects a good removal performance for a complex pharmaceutical compound. The lower performance with TCH is likely due to its bulky, multi-ring structure and functional groups (e.g., -OH, -NH_2_), which can hinder radical access or promote surface interactions that reduce degradation. Overall, the results confirm the wide applicability of the LM/PMS system for the degradation of diverse pollutants, with particularly high efficiency toward dyes and phenolic compounds, and antibiotics like TCH.

### Scavenging experiments

**Fig. 8:**
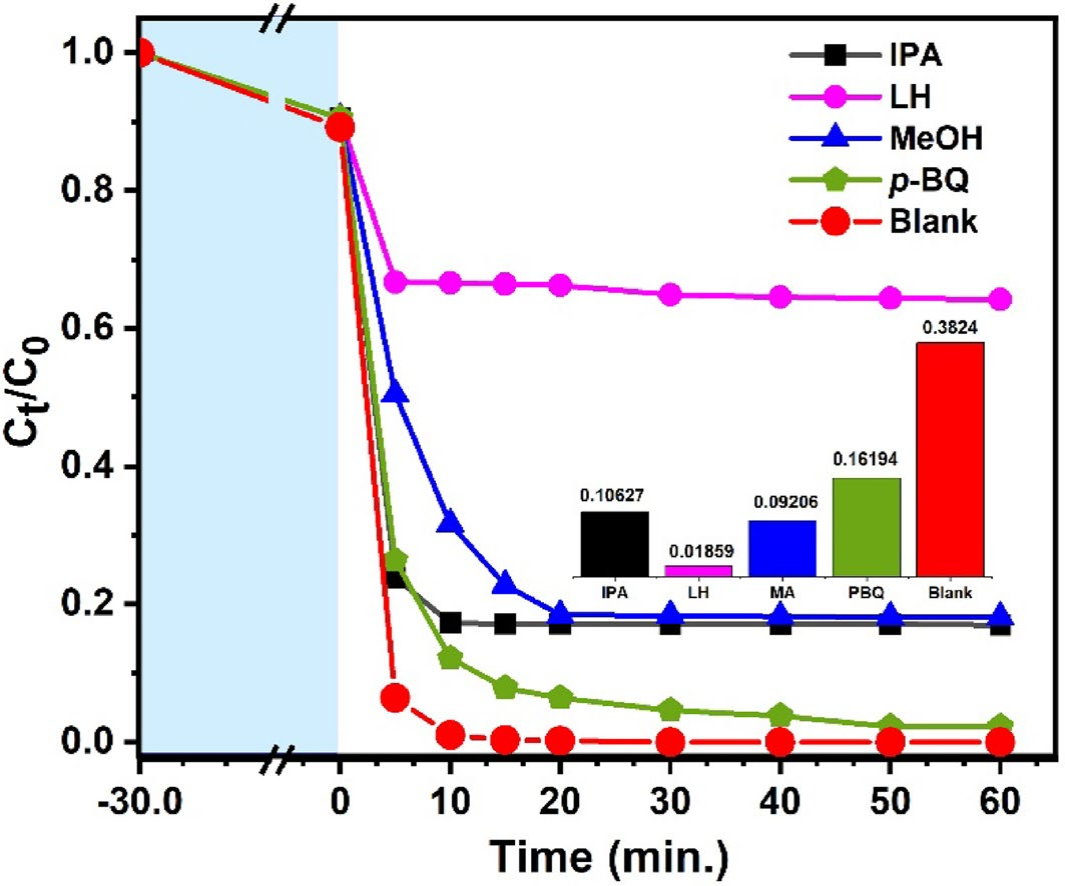
Scavenging Experiments at the optimum conditions.

The radical-scavenging tests were conducted to elucidate the ROS contributing to PMS activation, scavenging experiments were performed under optimal conditions (2.5 mg catalyst, 10 mg PMS, 50 mL of 20 mgL^− 1^ MB, pH 7) using various quenchers: (IPA, ^•^OH scavenger), (MeOH, SO_4_^•-^, and ^•^OH scavenger), (PBQ, O_2_^•-^ scavenger), and (LH, ^1^O_2_ scavenger) were introduced individually into the reaction system to elucidate the active species^62,73^. As illustrated in Fig. 8, the blank system (without scavenger) achieved nearly complete MB degradation within 10 min, exhibiting a pseudo-first-order with a rate constant (k = 0.3824 min^− 1^). The incorporation of scavengers significantly suppressed the degradation efficiency, confirming the involvement of multiple ROS. The strongest inhibition was observed with LH (k = 0.01859 min^− 1^), suggesting that singlet oxygen (^1^O_2_) acts as the primary reactive species in the system. Considerable decreases in reaction rate were also recorded in the addition of IPA (k = 0.10627 min^− 1^) and MeOH (k = 0.09206 min^− 1^), highlighting the contribution of hydroxyl (^•^OH) and sulfate (SO_4_^•-^) radicals. Meanwhile, moderate inhibition with PBQ (k = 0.16194 min^− 1^), confirmed the participation of superoxide radicals (O_2_^•-^) to a lesser extent. These results suggest that the degradation of MB proceeds through a dual oxidation mechanism that integrates both radical-mediated and non-radical routes. The non-radical pathway, primarily governed by singlet oxygen (^1^O_2_), governs the primary oxidation reactions, while ^•^OH, O_2_^•-^, and SO_4_^•-^ radicals act as auxiliary oxidants, enhancing the overall degradation efficiency.

**Fig. 9:**
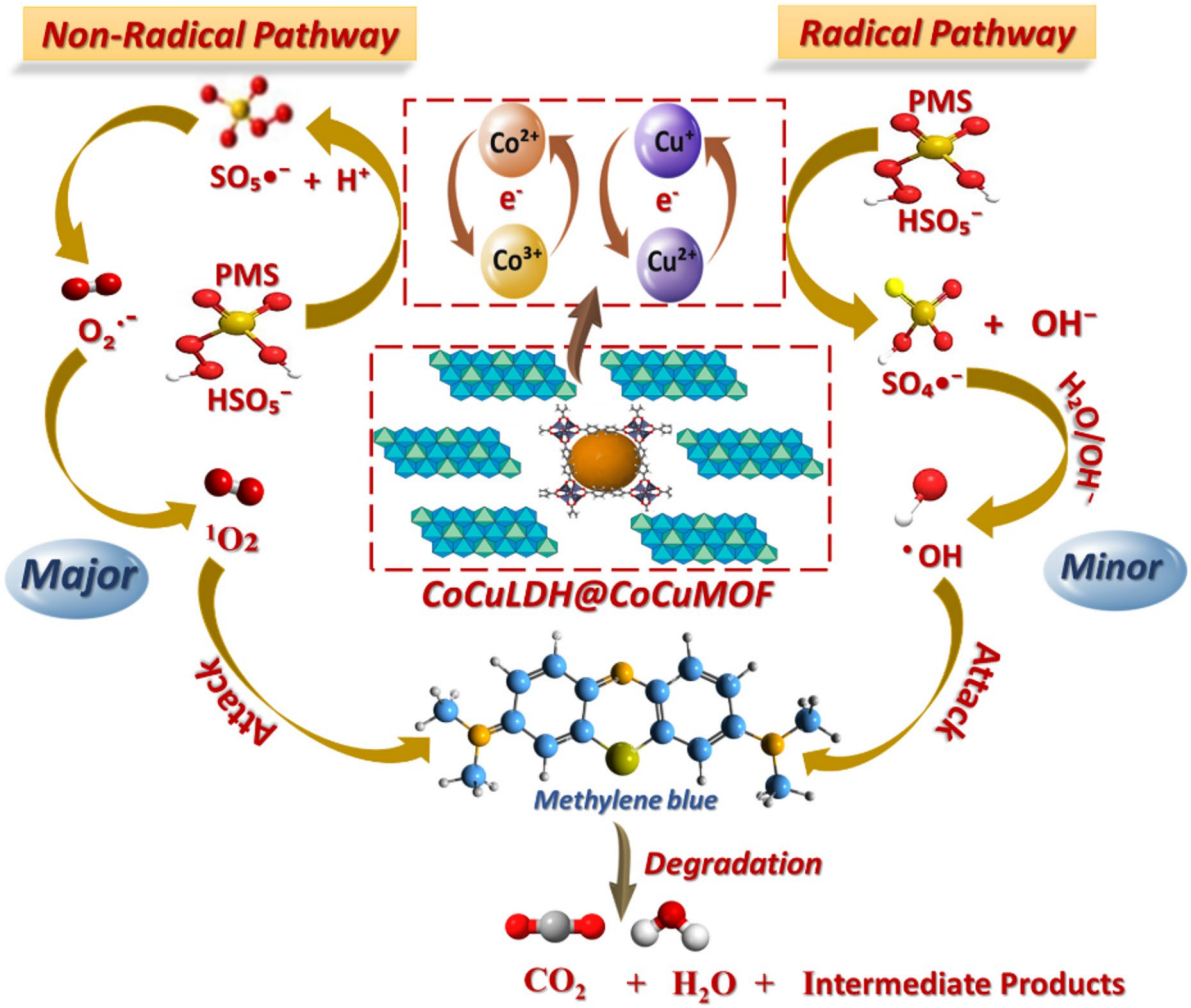
Mechanism of activation of PMS by CoCu@LDH@CoCuMOF (LM) for MB degradation.

The superior catalytic activity of the LM composite toward PMS activation arises from the synergistic interaction between Co and Cu redox centers, which simultaneously promote radical and non-radical ROS generation. The mechanistic radical and non-radical pathways is illustrated in Fig. 9. The bimetallic interface facilitates a continuous redox cycle between Co^2+^/Co^3+^ and Cu^+^/Cu^2+^ pairs^74^, activating PMS to yield sulfate radicals according to:

1 $${\mathrm{HSO}}_{5}^{ - } + {\text{ Co}}^{{2 + }} /{\text{ Cu}}^{ + } ~ \to {\text{ SO}}_{4}^{{ \bullet - }} + {\text{ Co}}^{{3 + }} {\text{ }}/{\text{ Cu}}^{{2 + }} {\text{ }} + {\text{ OH}}$$

The generated Co^3+^ and Cu^2+^ species are then reduced by PMS, maintaining the catalytic cycle:

2 $${\mathrm{HSO}}_{5}^{ - } + {\text{ Co}}^{{3 + }} {\text{ }}/{\text{ Cu}}^{{2 + }} {\text{ }} \to {\text{ SO}}_{5}^{{ \bullet - }} + {\text{ Co}}^{{2 + }} /{\text{ Cu}}^{ + } {\text{ }} + {\text{ H}}^{ + }$$

The generated SO_4_^•-^ radicals can subsequently react with water to produce hydroxyl radicals:

3 $${\mathrm{SO}}_{4}^{{ \bullet - }} + {\text{ H}}_{{\mathrm{2}}} {\text{O }} \to ^{ \bullet } {\text{OH }} + {\text{ H}}^{ + } {\text{ }} + {\text{ SO}}_{4}^{{ - 2}}$$

In parallel, the hybrid structure facilitates a direct PMS activation route leading to singlet oxygen (^1^O_2_) generation via recombination of SO_5_^•-^ intermediates^75^:

4 $${\mathrm{2SO}}_{{\mathrm{5}}} ^{{ \bullet - }} \to {\text{ 2SO}}_{4}^{{ - 2}} + ^{{\mathrm{1}}} {\mathrm{O}}_{{\mathrm{2}}}$$

Additionally, the abundant Lewis acid sites and high interfacial conductivity of the LM composite facilitate PMS uptake and support interfacial electron transfer between the dye and PMS–catalyst complex, enabling efficient oxidation even without free radicals in the bulk solution. Consequently, the LM catalyst activates PMS through a synergistic multi-pathway mechanism encompassing radical species (^**•**^OH, SO_4_^•-^, O_2_^•-^) and non-radical (^1^O_2_, electron transfer) routes. This dual activation route underpins the exceptional catalytic performance and stability of LM in PMS-based AOPs.

### Mineralization of MB dye (COD analysis)

**Fig. 10:**
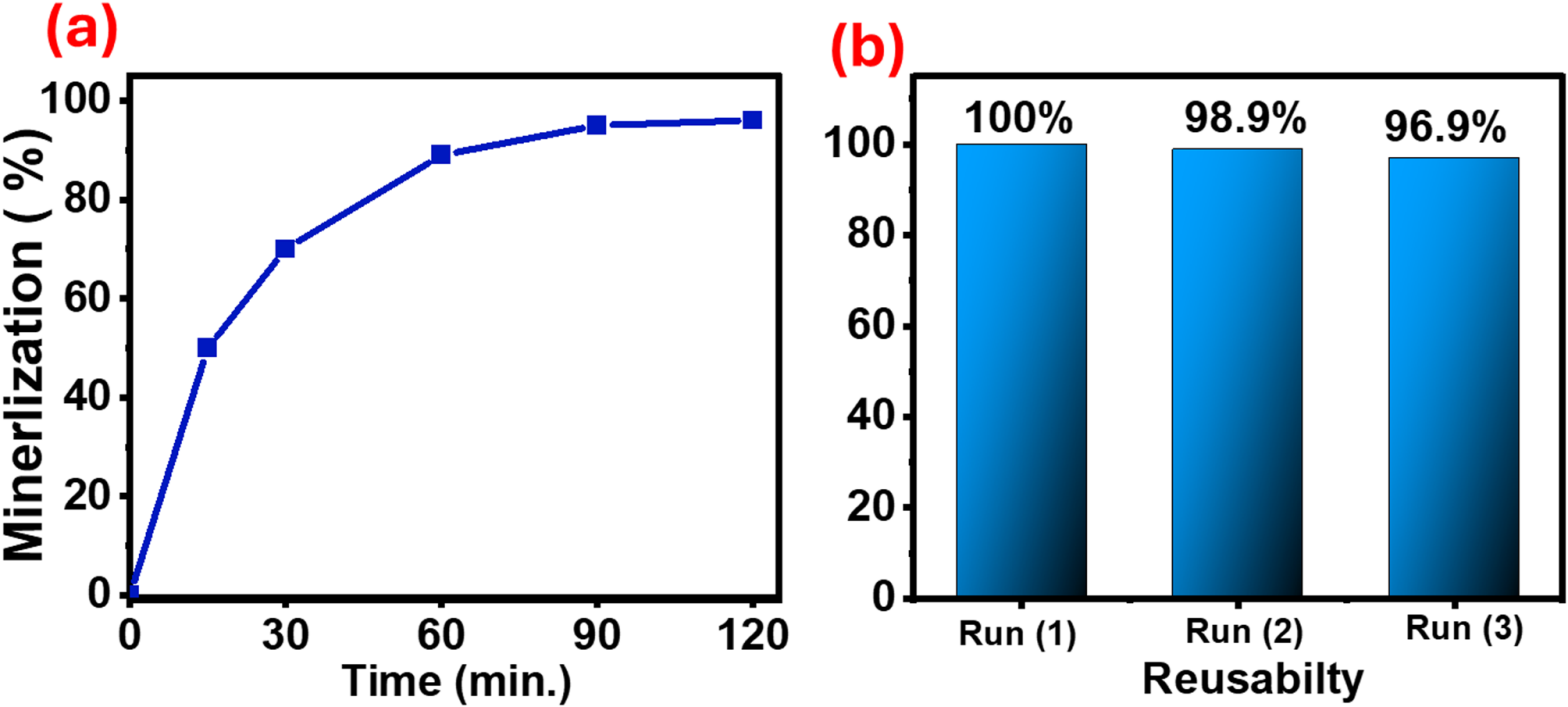
(**a**) Mineralization of MB (TOC analysis), (**b**) Reusability and recovery of LM for MB degradation.

The LM/PMS system efficiently mineralized MB, as evidenced by chemical oxygen demand (COD) analysis, showing continuous removal of organic carbon at intermediate reaction time points Fig. 10a. Under optimized conditions, approximately 96% mineralization was achieved, indicating not only rapid decolorization but also complete oxidative conversion to inorganic end-products (CO_2_ and H_2_O). These results highlight the system’s high catalytic efficiency and its strong potential for advanced wastewater treatment applications.

### The reusability

The stability of the LM catalyst was evaluated through three consecutive cycles of MB degradation under the optimized reaction conditions. After each run, the catalyst was recovered, washed, and reused. The degradation efficiencies remained at 100%, 98.9%, and 96.9% after the first, second, and third cycles, respectively, as illustrated in Fig. 10b.

The total efficiency loss of only ~ 3.9% after three consecutive cycles demonstrates good retention of catalytic activity and confirms the satisfactory reusability of the LM composite. To further assess structural stability, the used catalyst was characterized by XRD after the reuse experiment, as shown in Fig. S5a in the supplementary information. The diffraction pattern of the used catalyst closely resembles that of the fresh catalyst, with the main characteristic reflections of the LDH and MOF phases remaining unchanged even after the reduction of the crystallinity and partial structural disorder after PMS activation. No new diffraction peaks were detected, indicating that the LM heterostructure maintained its structural integrity during the catalytic process and that no obvious phase transformation occurred after repeated use. FTIR provides additional evidence that the main chemical structure was partly preserved as shown in Fig. S5b. The used sample retained the broad O-H band around 3400 cm^− 1^, the carboxylate bands near 1600 and 1400 cm^− 1^, and the low-wavenumber M-O/M-O-C bands. Therefore, the combined XRD and FTIR results suggest partial structural restructuring rather than complete catalyst collapse.

#### Leaching analysis and heterogeneous nature of catalysis

To further assess catalyst stability beyond reuse-cycle experiments, metal leaching from the LM composite was investigated by atomic absorption spectroscopy (AAS). In deionized water without PMS, the concentrations of dissolved Co and Cu were only 0.04 and 0.012 mg L^− 1^, respectively, indicating excellent stability of the catalyst in aqueous media under the optimum conditions. Furthermore, the LM catalyst retained high catalytic activity during three consecutive reuse cycles, with degradation efficiency decreasing only slightly from 98.9% in the first cycle to 96.9% after the third cycle, further demonstrating its good stability and reusability.

### Comparison with previously reported PMS-activated catalysts

To evaluate the catalytic performance of the LM composite in the context of current literature, a comparison with representative PMS-activated catalysts reported for MB degradation is presented in Table 1. The LM composite achieved 98.9% MB degradation within 10 min under near-neutral conditions and a relatively low catalyst dosage. Compared with the selected reported systems, the LM catalyst exhibits a high degradation rate together with good reusability, maintaining 96.9% degradation efficiency after three consecutive cycles. These results demonstrate the promising potential of the LM heterostructure for PMS-based wastewater treatment applications.

**Table 1:** Comparative performance of PMS-activated catalysts for MB degradation.

Catalyst/System	Reaction Conditions	Efficiency/Time	Reference
NiCo-LDH [1:2]	MB 10 mg L^− 1^; PMS 75 mg L^− 1^; Catalyst 75 mg L^− 1^; pH 7	97.2% in 12 min;	^76^
FeCu-MOF	MB 0.2 mM; PMS 6.0 mM; Catalyst 0.6 g L^− 1^; 25 °C	100% in 30 min	^77^
ZIF-9@CoAl-LDH (S40)	MB 10 mg L^− 1^; PMS 10 mg; Catalyst 5 mg; 100 mL solution	~ 97% in 30 min	^78^
Mn/Co composite oxide (Mn1Co2)	MB 10 mg L^− 1^; PMS 0.6 mM; Catalyst 0.04 gL^− 1^; 25 °C	94.16% in 25 min	^79^
Vacancy-strengthened Cu/Co oxide (CuCoO-2)	MB 20 mg L^− 1^(50 mL solution); PMS 0.5 g L^− 1^; Catalyst 0.5 g L^− 1^; 25 °C.	100% in 40 min	^80^
Cu@Co-MOFs	MB 0.2 mM; PMS 2.0 mM; Catalyst 0.1 g L^− 1^; 25 °C	100% in 30 min	^81^
FeCo-BDC MOF	MB 0.2 mM; PMS 1.0 mM; Catalyst 50 mg L^− 1^	100% in 15 min	^82^
CoFe₂O₄/ZIF-8 composite	MB 20 mg L^− 1^; PMS 0.3 g L^− 1^; Catalyst 0.3 g L^− 1^; 20 °C	97.9% in 60 min	^82^
CoCu-LDH@CoCu-MOF (LM)	MB 20 mg L^− 1^ (50 mL solution); PMS 10 mg; Catalyst 2.5 mg; pH 7; 25 °C	98.9% in 10 min	**This Work**

## Conclusion

This work demonstrates that integrating L with M into hybrid composites provides a highly efficient catalytic platform for PMS. Among the synthesized systems, LM exhibited the highest degradation efficiency, which is attributed to its optimized heterostructure, enhanced electron transfer capability, and abundant accessible reactive sites. Under optimal conditions, the catalyst achieved nearly complete degradation, with 98.9% removal of MB within only 10 min, confirming its exceptional catalytic activity and rapid PMS activation performance. The material also demonstrated efficient removal of a wide range of organic contaminants, including dyes, phenolic species, and antibiotics, confirming its broad applicability for wastewater purification. Despite partial inhibition by carbonate ions, LM maintained strong activity in the presence of other co-existing anions, highlighting its suitability for real wastewater environments. Moreover, the catalyst revealed remarkable recyclability, maintaining more than 96.9% of its degradation performance after three successive runs, indicating excellent structural stability and durability. Overall, this study provides valuable insights into the rational design of multifunctional LDH-MOF heterostructures and underscores their great potential for sustainable and large-scale environmental remediation applications.

## Supplementary Information

Below is the link to the electronic supplementary material.

Supplementary Material 1

## Data Availability

All data that support the findings of this study are included within the article (and any supplementary files).
