# Supplementary material for: Dual mechanisms of peroxymonosulfate activation by a CoCuLDH/CoCuMOF composite for pollutant degradation via radical and nonradical pathways
Source: Sci Rep. 2026 Aug 8;16:24544. doi: 10.1038/s41598-026-64251-y (PMC13452812; doi:10.1038/s41598-026-64251-y)
Supplement: Supplementary file 1 [file 41598_2026_64251_MOESM1_ESM.docx]

**Dual mechanisms of peroxymonosulfate activation by a CoCuLDH/CoCuMOF composite for pollutant degradation via radical and nonradical pathways**

Aya S. Mahmoud^a^ and Abdelrahman M. Rabie^b*^

^a^ Chemistry Department, Faculty of Women, Ain Shams University, Cairo, Egypt.

^b^ Petrochemicals department, Egyptian Petroleum Research Institute (EPRI), Nasr City 11727, Cairo, Egypt.

Corresponding authors: Aya S. Mahmoud; Abdelrahman M. Rabie
Email: [aya.sabry@women.asu.edu.eg](mailto:aya.sabry@women.asu.edu.eg); amrabie@epri.sci.eg
Mobile/ +201228289661

**S1. Characterization of the catalyst**

Information concerning the crystal structures of the synthesized samples was identified by powder XRD using a Philips diffractometer in the range 10°- 60° 2θ by Cu Ka radiation with Cu Kα radiation (λ=1.5406 Å). Fourier transform infrared (FTIR) spectra were recorded using a Nicolet iS10 spectrometer (Thermo Fisher Scientific) equipped with a deuterated triglycine sulfate (DTGS) detector. All spectra were collected in transmission mode using the KBr pellet technique (sample:KBr mass ratio = 1:100) over the wavenumber range of 4000-500 cm^-1^ with a spectral resolution of 4 cm^-1^. A total of 32 co-added scans were accumulated per spectrum to ensure an adequate signal-to-noise ratio. Background spectra were collected under identical conditions immediately prior to sample measurement and automatically subtracted. XPS measurements were conducted on an ESCALAB MK II system with Al Kα excitation (1487.6 eV Nitrogen physisorption isotherms (BET) were measured using a Micromeritics ASAP 2405 N V1.01 adsorption system at −196°C. Pore size distribution and surface area were calculated from the isotherms using the BJH and BET methods. The silver-coated materials were examined through the scanning electron microscope (SEM, 30 kV, model Jeol 5410). TEM images were taken using a Jeol 2010 DV 300W1 system operating at 200 kV. The analysis of MB concentration was investigated by a UV–vis spectrophotometer (6300 UV/Vis JENWAY) to evaluate the extent of the dye degradation.

**Fig.S1.** XPS survey spectrum of the ML composite showing characteristic peaks of Cu 2p, Co 2p, O 1s, and C 1s, confirming the presence of the expected elements in agreement with the high-resolution XPS analysis presented in the main manuscript.

**Table S1**: The textural properties of the prepared materials.

| Sample name | S_BET_  m^2^ g^-1^ | Total pore volume  cm^3^ g^-1^ | Pore diameter  nm |
| --- | --- | --- | --- |
| L | 50.61 | 0.008 | 3.935 |
| M | 24.69 | 0.00496 | 3.85 |
| ML | 18.59 | 0.00367 | 3.92 |
| LM | 46.26 | 0.00985 | 3.93 |

**Fig. S2.** EDX spectrum and elemental mapping images of the prepared CoCu-LDH@CoCu-MOF composite, showing the uniform distribution of Co, Cu, O, C, and N elements. The corresponding EDX table lists their atomic and weight percentages, confirming the successful incorporation of both metal species in the hybrid framework.

**Fig.S3:** UV-Vis spectra and removal efficiencies (inset) of MB under control conditions (MB only, MB + PMS, MB + LM, MB + LM + PMS, and MB + LM + PMS in the dark), confirming that MB degradation proceeds via chemical PMS activation rather than adsorption or photocatalysis

**Fig. S4.** Linear fitting of the pseudo-first-order kinetic model (ln(C₀/Ct) vs. time) for MB degradation over the LM catalyst under various operating conditions: (a) comparison of the kinetic performance of all catalysts (L, M, LM, ML); (b) effect of catalyst dosage; (c) effect of PMS dosage; (d) influence of solution pH; and (e) effect of coexisting ions, and (f); Scavenging experiments at the optimum conditions. In all cases, the linear correlation confirms that MB degradation over the CoCu-LDH@CoCu-MOF (LM) catalyst follows pseudo-first-order kinetics.

**Fig. S5.** Comparison of the XRD patterns of the fresh and reused LM catalyst. The retained characteristic diffraction reflections indicate the structural stability of the LDH/MOF heterostructure during repeated catalytic operation**.**
